# Hemichorea associated with non-ketotic hyperglycemia: a case report and literature review

**DOI:** 10.3389/fcdhc.2026.1746146

**Published:** 2026-04-24

**Authors:** Zhou Liu, Juan Chen, Chao Gui Zhang, Yi Ming Xiang, Ao Lin Zhuang, Yi Li, Jing Jin Man, Gui You Li, Min Jie Fan, Hui Li

**Affiliations:** 1 Department of Medical, Hubei Minzu University, Enshi, China; 2 Department of General Medicine, The Central Hospital of Enshi Tujia and Miao Autonomous Prefecture, Enshi, China; 3 Department of Neurology, Minda Hospital of Hubei Minzu University, Enshi, China

**Keywords:** clinical manifestations, early diagnosis, hemichorea, imaging, non-ketotic hyperglycemia

## Abstract

Hemichorea associated with non-ketotic hyperglycemia (HC-NH) is a rare neurological complication and is also considered a specific complication of diabetes. Its exact pathogenesis remains unclear. We report a case of a 79-year-old female patient with HC-NH. Her primary clinical presentation included involuntary movements in the right limbs that began 3 days prior to admission. These movements worsened during emotional agitation and could be accompanied by insomnia but resolved after falling asleep. Imaging studies revealed no significant intracranial lesions on CT. MRI showed hyperintensity on T1-weighted images (T1WI) in the left globus pallidus and putamen and slightly lower signal intensity on T2-weighted imaging (T2WI). Fingerstick glucose monitoring during hospitalization showed only mild elevation, and urine ketones showed negative results. Treatment included glucose control, tiapride, Trihexyphenidyl, and haloperidol for choreiform movements, along with lorazepam to alleviate anxiety and tension. The patient showed slight improvement, and choreiform movements markedly diminished 3 months after discharge. This case demonstrates that blood glucose levels may not be markedly elevated at the onset of HC–NH, potentially leading to delayed or misdiagnosis. Additionally, symptom control may not achieve optimal results in the short term. Therefore, early diagnosis and treatment are crucial for the prognosis of this condition.

## Introduction

Hemichorea associated with non-ketotic hyperglycemia (HC-NH) is a rare neurological complication predominantly observed in patients with uncontrolled diabetes mellitus, particularly elderly women. It is characterized by acute or subacute onset of unilateral or bilateral choreiform movements, often associated with hyperglycemia without ketoacidosis. In recent years, with the rising incidence of diabetes and advancements in imaging technology, reports of this condition have gradually increased. However, due to its atypical clinical manifestations, it is prone to misdiagnosis or missed diagnosis (1, 2). This article presents a typical case report accompanied by a literature review, with the dual objectives of improving clinicians’ comprehension of this disease and facilitating early diagnosis and prompt treatment.

## Case presentation

A 79-year-old female patient presented to the Neurology Department on January 7, 2025, due to “abnormal movements of the right limbs for 3 days”. Past medical history included diabetes mellitus diagnosed 2 months prior, with previous (unspecified) medication discontinued by the patient herself. She also had a 20-year history of hypertension, currently managed with regular oral nifedipine sustained-release tablets. Denies history of psychiatric disorders and family history of movement disorders. Three days prior to admission, without obvious precipitating factors, the patient developed abnormal movements of the right limbs, manifesting as involuntary, irregular motions, including facial grimacing, accompanied by discomfort in the lower back and right knee joint. Symptoms worsened with emotional agitation and disappeared during sleep. Physical examination showed the following findings: blood pressure, 143/80 mmHg; other vital signs, normal. Cardiopulmonary and abdominal examinations revealed no significant positive findings. Neurological examination showed the following findings: involuntary, irregular choreiform movements of the right limbs. Muscle strength and tone in all limbs were normal. Right Babinski sign was equivocal. No other significant positive neurological signs were noted.

## Investigations

**Figure 1:**
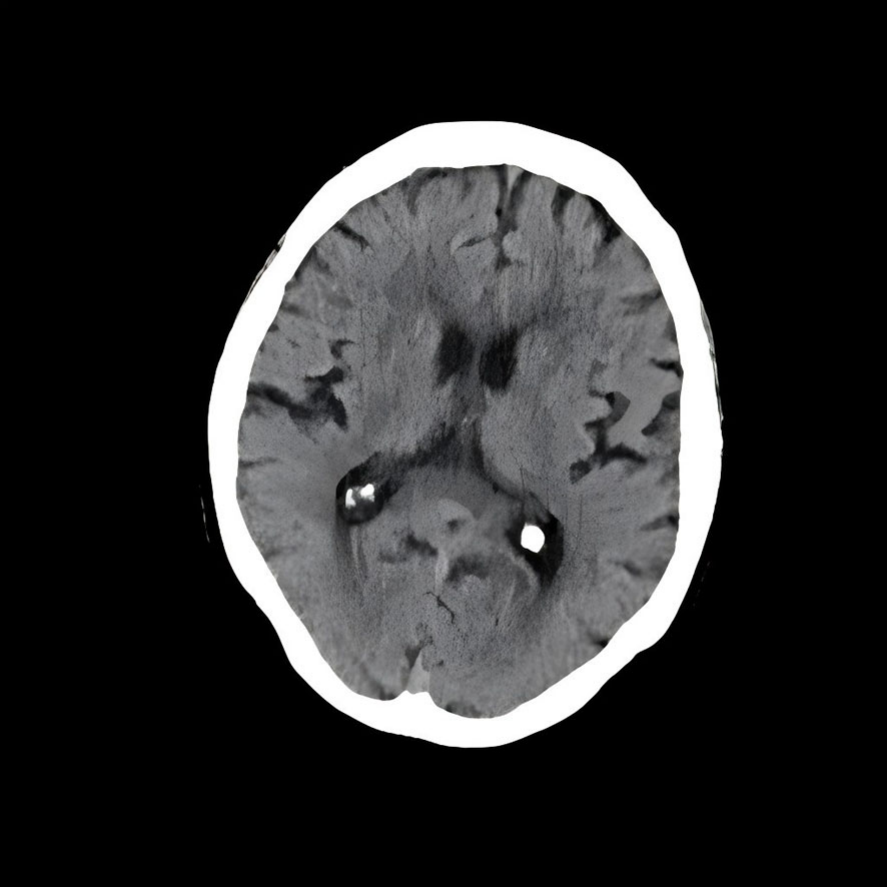
CT scan: Multiple cystic and patchy hypodense lesions in bilateral basal ganglia, corona radiata, and centrum semiovale. Reduced white matter density adjacent to bilateral lateral ventricles. Clear gray–white matter differentiation.

Cranial CT: Multiple cystic and patchy hypodense lesions in bilateral basal ganglia, corona radiata, and centrum semiovale. Reduced white matter density adjacent to bilateral lateral ventricles. Clear gray–white matter differentiation (**Figure 1**).

**Figure 2:**
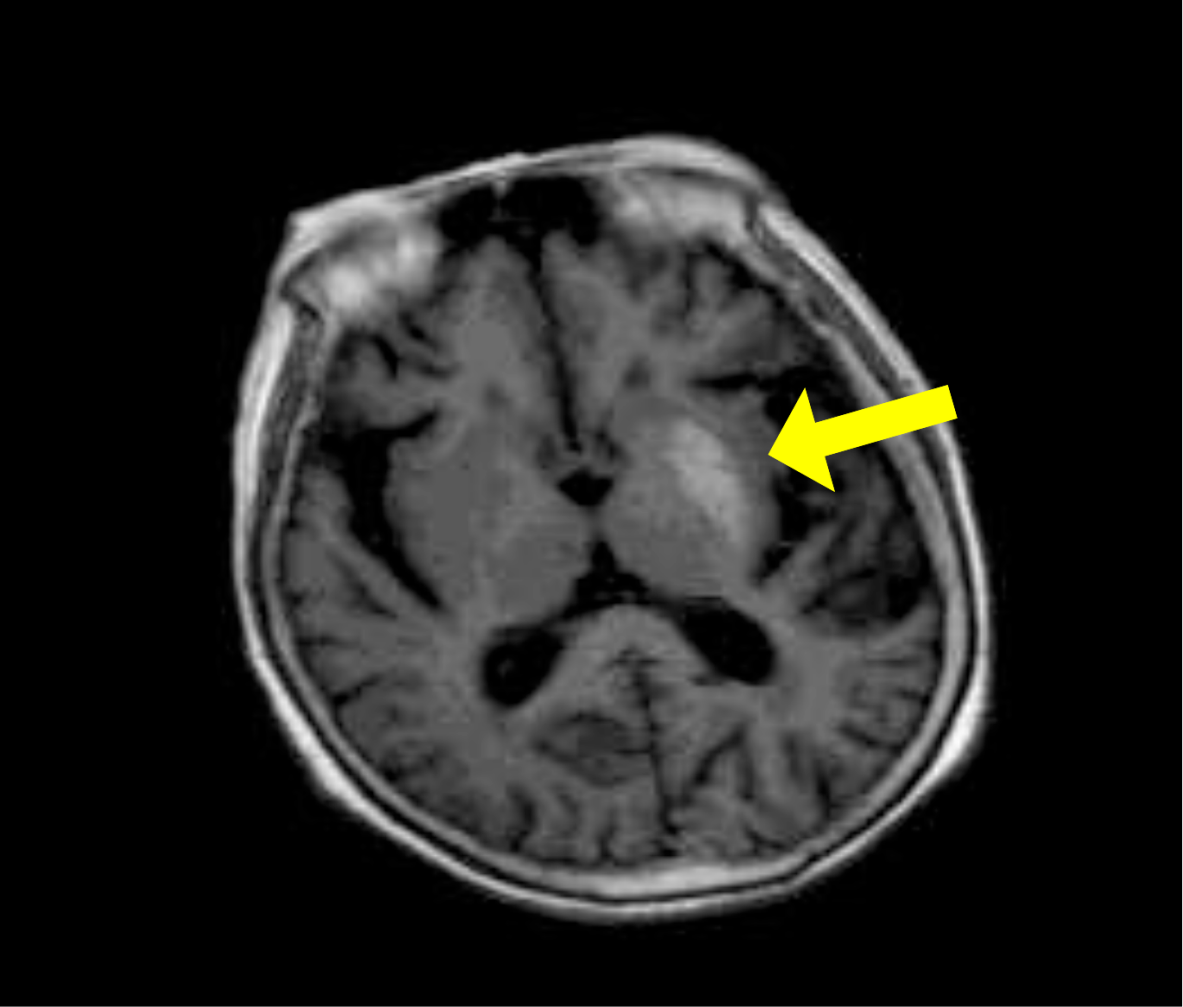
T1-weighted images (T1WI): Patchy slightly hyperintense signal primarily in the left basal ganglia (globus pallidus and putamen) (indicated by yellow arrow).

**Figure 3:**
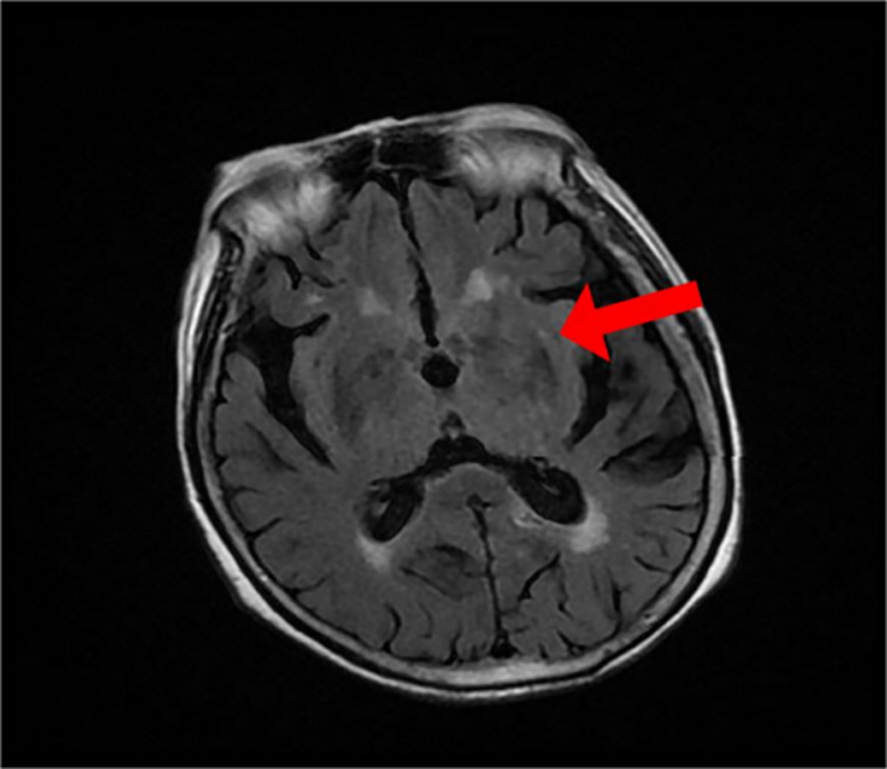
T2-weighted imaging (T2WI): Corresponding area shows slightly hypointense signal (indicated by red arrow), visible high signal in the brain white matter.

**Figure 4:**
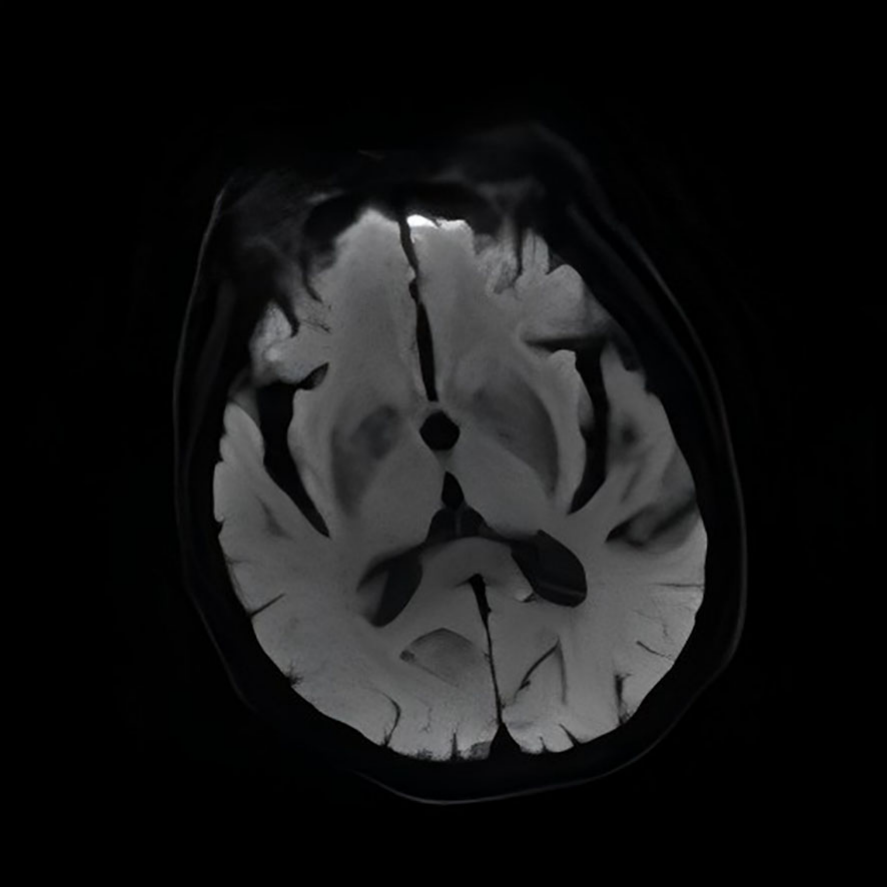
Diffusion-weighted imaging (DWI): No significant restricted diffusion in the brain parenchyma.

Cranial MRI: Patchy slightly hyperintense signal on T1WI primarily in the left basal ganglia (globus pallidus and putamen) (**Figure 2**). The corresponding area showed a slightly hypointense signal on T2WI (**Figure 3**). Diffusion-weighted imaging (DWI) showed no significant restricted diffusion in the brain parenchyma (**Figure 4**).

Electrocardiogram: Normal.

Fasting blood glucose: 8.66 mmol/L.

Glycated hemoglobin (HbA1c): 15.5%.

Renal function: urea: 14.22 mmol/L, creatinine: 134.6 µmol/L.

Urinalysis: urine glucose +/−, ketones negative.

Based on clinical manifestations, past medical history, and various examinations, chorea induced by stroke was excluded, and diseases such as parasitic infections were also not considered. A cerebral metabolic disorder was considered, with a preliminary diagnosis of HC-NH. Treatment was initiated with oral tiapride to control choreiform symptoms, and intravenous Xueshuantong and citicoline to improve circulation and provide neuroprotection. After 2 days of treatment, the patient’s limb movements showed no significant improvement. Trihexyphenidyl 1 mg orally three times daily and haloperidol 10 mg intramuscularly at night were added for symptom control. Vildagliptin 50 mg orally before breakfast was started for glycemic control. During hospitalization, fingerstick blood glucose monitoring showed fasting levels between 5 and 8.8 mmol/L and postprandial levels between 4.4 and 10.7 mmol/L. Symptom improvement remained minimal, and the patient developed anxiety, tension, and insomnia. The haloperidol regimen was gradually adjusted to 2 mg orally every 8 h, and lorazepam 1 mg orally nightly was added. Subsequently, the patient’s limb movements gradually improved. The clinical course of the patient from symptom onset to 6-month follow-up is clearly documented in the timeline table (**Table 1**), which records the key events including the onset of right limb involuntary choreiform movements on January 4, 2025, admission on January 7, 2025, sequential adjustment of treatment regimens during hospitalization, and progressive symptomatic improvement at 1, 3, and 6 months after discharge. At the 1-month follow-up after discharge, the patient still had right limb movements, albeit with slightly reduced amplitude. Telephone follow-ups at 3 and 6 months revealed significant symptomatic improvement, but the patient declined to return for an in-person follow-up visit.

**Table 1:** Timeline Table.

Time point	Clinical events and interventions
January 4, 2025	Onset of involuntary choreiform movements in the right limbs; no obvious trigger
January 7, 2025	Admission to the Department of Neurology; initial evaluation performed
During admission	Cranial CT, MRI, laboratory tests (glucose, HbA1c, renal function, urinalysis); diagnosis: HC-NH
Days 1–2 after admission	Initial treatment: tiapride + Xueshuantong + citicoline + vildagliptin; no obvious improvement
Day 3 after admission	Added trihexyphenidyl (1 mg tid) and intramuscular haloperidol (10 mg qn); minimal improvement
During late hospitalization	Adjusted haloperidol to 2 mg q8h orally; added lorazepam (1 mg qn); symptoms gradually improved
Discharge	Stable condition; continued oral medications for glycemic control and choreic symptoms
1 month after discharge	Follow-up: right limb movements persisted with reduced amplitude
3 months after discharge	Telephone follow-up: choreiform movements markedly diminished
6 months after discharge	Telephone follow-up: significant symptomatic improvement; patient declined in-person re-examination

## Discussion

HC-NH is a rare neurological complication most commonly seen in patients with long-standing uncontrolled diabetes mellitus, particularly elderly women, with an average age of onset around 71 years. The estimated prevalence is less than 1 in 100,000 (3). The pathogenesis is not fully elucidated but likely involves a combination of hyperglycemic metabolic derangement, ischemia, and microhemorrhage. The characteristic MRI findings of T1 hyperintensity and reduced ADC values in the basal ganglia suggest possible microhemorrhage and ischemia, respectively (4). Postmortem brain examinations in HC-NH cases have revealed astrocytosis, hemosiderin deposits outside blood vessels, and iron deposits on penetrating vessels in the posterior putamen, supporting the presence of microhemorrhage (5). Hyperglycemia can increase blood viscosity and impair local microcirculation, leading to ischemia and metabolic abnormalities in the basal ganglia. Furthermore, hyperglycemia may disrupt neurotransmitter balance in the basal ganglia, particularly affecting GABAergic neuronal metabolism (6), thereby triggering choreiform movements. A SPECT study demonstrated significantly reduced blood flow in the contralateral striatum to the affected limbs in HC-NH patients compared with controls, indicating striatal hypofunction (7). One case report even documented a patient with HC-NH progressing to acute ischemic stroke in the ipsilateral basal ganglia (8). Other reports suggest a possible association with acanthocytosis (9). Chorea-acanthocytosis(ChAc) is caused by mutations in the VPS13A gene on autosome 9q21. The gene encodes the protein chorein, which plays a key role in maintaining cytoskeletal stability, membrane fluidity, and lipid transport. This protein is highly expressed in all regions of the brain and on red blood cell membranes. (10, 11). Diabetes (especially non-ketotic hyperglycemia) can trigger diabetic striatopathy (CHBG), leading to acute choreic movements, which resemble the choreic symptom phenotype of ChAc (12) .Although existing studies have not directly verified the direct effects of hyperglycemia on chorein function, pathways revealed through yeast models, such as calcium signaling, copper and iron homeostasis, sphingolipid metabolism, and the cytoskeleton, provide important clues for understanding the mechanistic overlap between diabetes and ChAc. These findings suggest that in ChAc patients with concurrent hyperglycemia, dual disruption of these pathways may exacerbate disease progression, which deserves further validation in higher-level models (such as neurons differentiated from patient-derived induced pluripotent stem cells) (13). A meta-analysis by Chua et al. found high average serum glucose and HbA1c levels at diagnosis (1), which differs slightly from our case where only HbA1c was markedly elevated, whereas monitored blood glucose levels were not significantly high. This discrepancy might be related to antecedent severe hyperglycemia.

Diagnosis of HC-NH relies primarily on clinical presentation, laboratory findings, and imaging characteristics. The typical presentation is acute or subacute onset of unilateral or bilateral choreiform movements associated with hyperglycemia but without ketoacidosis. The characteristic finding on brain MRI is T1-weighted hyperintensity in the basal ganglia (14), most commonly in the putamen, potentially related to local calcification, mineralization, or hemorrhage. Differential diagnoses include Huntington’s disease, Wilson’s disease, and Sydenham’s chorea.

Regarding treatment, the primary measure is aggressive glycemic control, often achieved with insulin. For choreiform movements, dopamine receptor antagonists such as haloperidol or risperidone can be used (1). Most patients have a favorable prognosis with timely treatment, and symptoms may resolve completely within weeks to months. However, some patients may experience residual mild movement disorders or recurrence (15). Reports exist of rapid symptom improvement with strict insulin control and haloperidol, with no recurrence during the 1-year follow-up (4), which contrasts somewhat with our case where significant improvement occurred gradually over 3 months post-discharge. Hamed and colleagues found that factors such as white matter lesions, hypertension, and blood-brain barrier damage can reduce basal ganglia functional reserve. Individuals with white matter hyperintensities are more prone to basal ganglia lesions, and even after imaging shows reversibility, movement disorders persist, suggesting irreversible neuronal damage (16). This provides a mechanistic reference for understanding how factors like white matter lesions and hypertension delay the recovery of chorea symptoms in diabetic striatal disease.

A limitation of this case is that susceptibility-weighted imaging (SWI) was not performed during hospitalization to further confirm the presence of microhemorrhage in the intracranial lesion. Furthermore, follow-up after discharge was limited to telephone contact; the patient declined to return for repeat brain MRI, preventing assessment of lesion resolution, and specific post-discharge blood glucose values were not obtained.

In conclusion, HC-NH is a rare neurological complication of diabetes mellitus. Clinicians should be aware of this condition, as early diagnosis and treatment are crucial for prognosis.

## Data Availability

The datasets presented in this article are not readily available because of ethical and privacy restrictions. Requests to access the datasets should be directed to the corresponding authors.

## References

[1] Chua CB Sun CK Hsu CW Tai YC Liang CY Tsai IT . Diabetic striatopathy": clinical presentations, controversy, pathogenesis, treatments, and outcomes. Sci Rep. (2020) 10:1594. doi: 10.1038/s41598-020-58555-w. PMID: 32005905 PMC6994507

[2] Shafran I Greenberg G Grossman E Leibowitz A . Diabetic striatopathy-does it exist in non-Asian subjects? Eur J Intern Med. (2016) 35:51–4. doi: 10.1016/j.ejim.2016.05.026. PMID: 27296589

[3] Bendi VS Matta A Torres-Russotto D Shou J . Bilateral chorea/ballismus: detection and management of a rare complication of non-ketotic hyperglycaemia. BMJ Case Rep. (2018) 2018:bcr2018224856. doi: 10.1136/bcr-2018-224856. PMID: 29925556 PMC6011499

[4] Wang W Tang X Feng H Sun F Liu L Rajah GB . Clinical manifestation of non-ketotic hyperglycemia chorea: a case report and literature review. Med (Baltimore). (2020) 99:e19801. doi: 10.1097/md.0000000000019801. PMID: 32481362 PMC12245323

[5] Mestre TA Ferreira JJ Pimentel J . Putaminal petechial haemorrhage as the cause of non-ketotic hyperglycaemic chorea: a neuropathological case correlated with MRI findings. J Neurol Neurosurg Psychiatry. (2007) 78:549–50. doi: 10.1136/bcr.08.2008.0785. PMID: 17435197 PMC2117816

[6] Chen C Zheng H Yang L Hu Z . Chorea-ballism associated with ketotic hyperglycemia. Neurol Sci. (2014) 35:1851–5. doi: 10.1007/s10072-014-1968-1. PMID: 25262066

[7] Chang MH Li JY Lee SR Men CY . Non-ketotic hyperglycaemic chorea: a SPECT study. J Neurol Neurosurg Psychiatry. (1996) 60:428–30. doi: 10.1136/jnnp.60.4.428. PMID: 8774410 PMC1073898

[8] Carrion DM Carrion AF . Non-ketotic hyperglycaemia hemichorea-hemiballismus and acute ischaemic stroke. BMJ Case Rep. (2013) 2013:bcr2012008359. doi: 10.1136/bcr-2012-008359. PMID: 23470671 PMC3618727

[9] Pisani A Diomedi M Rum A Cianciulli P Floris R Orlacchio A . Acanthocytosis as a predisposing factor for non-ketotic hyperglycaemia induced chorea-ballism. J Neurol Neurosurg Psychiatry. (2005) 76:1717–9. doi: 10.1136/jnnp.2005.067033. PMID: 16291901 PMC1739427

[10] Peikert K Danek A . VPS13 Forum Proceedings: XK, XK-related and VPS13 proteins in membrane lipid dynamics. Contact (Thousand Oaks (Ventura County Calif)). (2023) 6:25152564231156994. doi: 10.1177/25152564231156994. PMID: 37366410 PMC10243564

[11] Kamińska J Kolakowski D . Proteins from Vps13 family: from molecular function to pathogenesis of neurodegenerative disorders. Postepy Biochemii. (2018) 64:275–87. doi: 10.18388/pb.2018_141, PMID: 30656912

[12] Chen X Ma C Zhi L Wei X Luo J Liang C . Hemichorea associated with nonketotic hyperglycemia. J Clin Endocrinol Metab. (2023) 108:e550–6. doi: 10.1210/clinem/dgad077. PMID: 36800278

[13] Kaminska J Soczewka P Rzepnikowska W Zoladek T . Yeast as a model to find new drugs and drug targets for VPS13-dependent neurodegenerative diseases. Int J Mol Sci. (2022) 23:5106. doi: 10.3390/ijms23095106. PMID: 35563497 PMC9104724

[14] Homaida M Kanodia AK Young N Yu WM . Diabetic striatopathy: a rare condition and diagnostic dilemma. BMJ Case Rep. (2021) 14:e240141. doi: 10.1136/bcr-2020-240141. PMID: 33452065 PMC7813318

[15] Chang X Hong W Yu H Yao Y . Chorea associated with nonketotic hyperglycemia: a case report with atypical imaging changes. Med (Baltimore). (2017) 96:e8602. doi: 10.1097/MD.0000000000008602, PMID: 29137086 PMC5690779

[16] Hamed S Mohamed K Elhameed SA Moussa E Abozaid H Lang A . Movement disorders due to selective basal ganglia lesions with uremia. Can J Neurological Sci Le J Canadien Des Sci Neurologiques. (2020) 47:350–65. doi: 10.1017/cjn.2020.29. PMID: 32051045

